# Exploring Protein Fold Space

**DOI:** 10.3390/biom10020193

**Published:** 2020-01-27

**Authors:** William R. Taylor

**Affiliations:** Francis Crick Institute, London NW1 1AT, UK; william.taylor@crick.ac.uk

**Keywords:** protein fold-space, protein structure comparison, secondary structure lattice

## Abstract

The model of protein folding proposed by Ptitsyn and colleagues involves the accretion of secondary structures around a nucleus. As developed by Efimov, this model also provides a useful way to view the relationships among structures. Although somewhat eclipsed by later databases based on the pairwise comparison of structures, Efimov’s approach provides a guide for the more automatic comparison of proteins based on an encoding of their topology as a string. Being restricted to layers of secondary structures based on beta sheets, this too has limitations which are partly overcome by moving to a more generalised secondary structure lattice that can encompass both open and closed (barrel) sheets as well as helical packing of the type encoded by Murzin and Finkelstein on small polyhedra. Regular (crystalline) lattices, such as close-packed hexagonals, were found to be too limited so pseudo-latticses were investigated including those found in quasicrystals and the Bernal tetrahedron-based lattice that he used to represent liquid water. The Bernal lattice was considered best and used to generate model protein structures. These were much more numerous than those seen in Nature, posing the open question of why this might be.

## 1. Protein Fold Space

### 1.1. Early Explorers

The idea that protein chains fold progressively by first forming secondary structure elements (SSEs) that then assemble into the native fold was introduced in the early 1970s by Ptitsyn and co-workers [1,2]. For the all-helical protein myoglobin, they demonstrated that this simple approach could generate a limited number of folds including the native, in which the hydrophobic residues were buried in a core. Following this rough initial assembly, or molten globule, it was then assumed that the chain and side-chains could search their local environment to finally adopt the exact close packing observed in native structures. Given only a few or several secondary structures, this secondary structure accretion model provided an attractive explanation for protein folding. However, as the number of secondary structures increased, their possible arrangements seemed to rise greatly, and using hydrophobic burial as the only selection criterion would not have sufficient power to limit the solutions. 

By the later 1970s, the number of known protein structures had increased sufficiently to allow patterns of preferred structure to emerge from a comparative analysis. Following the pioneering work of Nagano [3], Sternberg and Thornton [4] focused on the local organisation of beta strands and alpha helices (super-secondary structure) and particularly on the chiral preference of their connectivity. At that time, the amazing alpha/beta barrel structure of triosephosphate isomerase (TIM) had recently been solved by the Phillips group [5], and it looked increasingly plausible that local packing rules of secondary structure might be enough to explain the overall folds of this structure and that of the Rossmann fold observed in a dehydrogenases and some other structures known at that time [6]. Independently, Jane Richardson [7,8] had also been looking at patterns of secondary structure connectivity and enumerated possible beta strand arrangements allowed under the constraint of preserving connection handedness and other constraints. 

**Figure 1:**
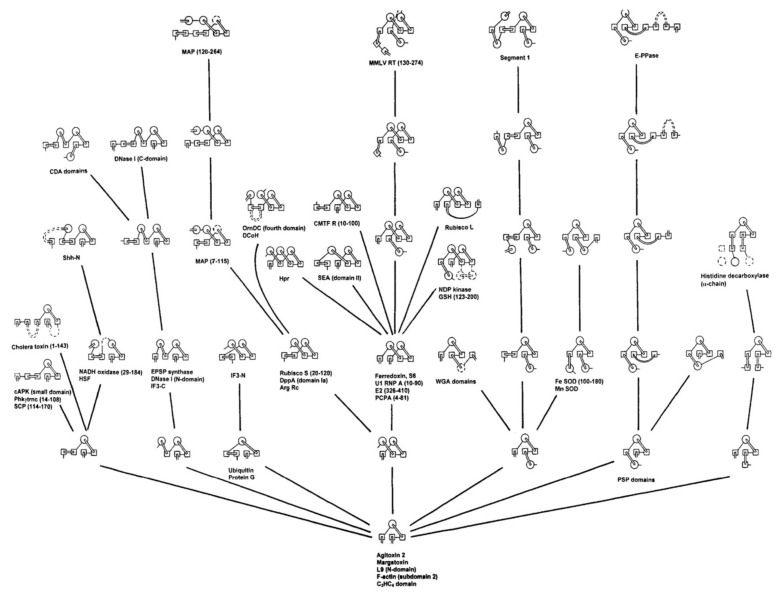
Efimov’s tree for some beta/alpha proteins. From a small nucleus (bottom), known structures were generated by the progressive addition of secondary structure elements.

Combining these beta-structure-based constraints along with the Ptitsyn model of SSE accretion, Efimov [9,10] showed, in the 1990s, that all the known folds across the three major classes of protein structure (all-alpha, all-beta and alpha/beta) could be rationalised by this approach (Figure 1). By showing that the then known protein “fold-space” could be accounted for in this way suggested strongly that proteins must be following a similar process in their folding. However, the simple model that the formation of a hydrophobic core would drive and guide the process did not appear to be sufficient. In work by Finkelstein and Reva [11], the formation of a hydrophobic core was formulated in a more rigorous model to generate a self-consistent “hydrophobic field”, and while this produced encouraging results when tested on a fixed lattice, the extra freedom introduced with an evolving lattice proved difficult.

### 1.2. Visualising Protein Fold-Space

Although the development of the approach pioneered by Ptitsyn and co-workers did not result in a definitive “solution” to the protein folding problem, it produced a powerful way of looking at how protein structure is organised, culminating in the comprehensive fold-trees of Efimov in the late 1990s. While Efimov’s “trees” do not (necessarily) imply any evolutionary relationship among the successive steps, they set out a clear hierarchical organisation, starting from a few packed secondary structures, which builds in simple steps up to the most complex structures (strictly, domains) that had been observed at that time. This hierarchical approach to protein fold organisation paved the way for the development of the more comprehensive collections found in the SCOP and CATH databases, but faced with the challenge of simply classifying the current known folds, these databases maintain only a simple hierarchy of a few levels with little attempt made at establishing any theoretical framework to relate folds within a level. 

**Figure 2:**
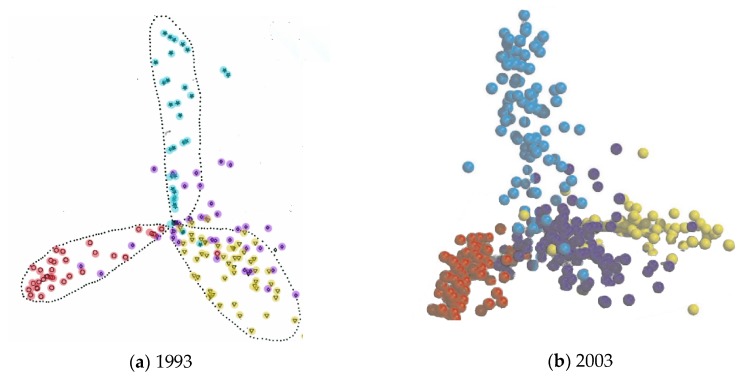
Protein fold-space projections. The distances among known structures are visualised by projection into Euclidean space. The structures (dots) are coloured according to structural class: red = alpha, yellow = beta, blue = alpha/beta and purple = alpha+beta. (The latter being a smaller less well-defined class). (**a**) As seen by Orengo et al. (1993) [14] and (**b**) as seen by Hou et al. (2003) [16]. Note the overall structure has remained unchanged over ten years.

The more automatic classifications, such the CATH and FSSP databases, do not rely on any underlying theory of protein structure but rather reflect their origins in the pairwise comparison of structures by the SSAP [12] and DALI [13] methods, respectively. For related folds, a degree of similarity can be based on their RMSD or equivalent measure, which can provide a metric to construct a dendrogram of the relationship among the structures. Applied to all pairs, this produces a matrix of distances which can be embedded (or projected) into a 3 or 2 dimensional Euclidean space thus giving a visualisation of the whole fold-space. Originally carried out using the SSAP program [14], this revealed a space divided into three lobes, reflecting the three dominant classes as previously identified by Levitt and Chothia [15]. However, as comparisons among quite different structures is fairly meaningless (typically random), then the majority of values in the matrix of distances will simply be large random values which contribute little to the fine structure of the space but simply serve to “repel” the different classes, giving the tri-lobed appearance. This explains why much the same structure was still found ten years later [16] (Figure 2).

## 2. Protein Topology

### 2.1. Topological Metrics

The reason why the classification databases have little internal structure is because their measures of pairwise similarity are essentially geometric and not topological. In the context of protein structure, the use of these terms requires some explanation. The comparison of protein structure by RMSd-based superposition is based on rigid 3D Euclidean geometry. Some measures, such as the distance based RMSD or the scoring function in the SSAP program, do allow some degree of “distortion”, allowing similarity to be detected among regions that have different displacements or orientations—for example, where two matching pairs of domains adopt a different relative juxtaposition in the two structures. By contrast, the term “topology”, although still a geometric concept, allows for similarity under extreme, indeed all possible, distortions in Euclidean space. For example, two circular pieces of string remain identical no matter how tangled each one has become (providing they are not cut and re-joined as a knot). However, in this true topological sense, protein (backbone) chains are very uninteresting—being all open strings and, hence, all identical. 

To capture the essence of a protein fold in a rigorous way requires a topological approach that is not based solely on the protein backbone chain. The introduction of cross-links, as found in disulphide-linked chains introduces true topological distinctions, creating closed circuits in the chain, which can even be linked as in cysteine-knots. While disulphide bonds are a relatively restricted feature, a more general source of cross-links can be found by considering hydrogen bonds. Local H-bonds, as in an alpha helix, create little discrimination but bonds between sequentially remote (non-local) parts of the chain provide a way to specify a fold. The comparison of H-bond networks was one of the factors contributing to the similarity of two structures in an early version of the SSAP program [17]; however, the importance of this factor in comparing distantly related or even proteins with a different fold was never fully investigated. While attractive, H-bond networks have some limitations. They are weak and are not always conserved between even closely related folds but more importantly, H-bond circuits in a network are essentially a form of distance matrix and so do not capture chirality.

### 2.2. Topology Diagrams

The 2 dimensional diagrams used by Nagano, Sternberg, and Thornton, Ptitsyn, Finkelstein, and others capture some of the topological requirements discussed above. Being dominated by beta sheet structure, they are essentially based on non-local H-bond interactions with the advantage that most beta strands involve several H-bonds and so are less susceptible to the “random” evolutionary structural variations seen within a family of homologous proteins. Despite being 2D, chirality is still included through the implicit third dimension along the secondary structure axis (running perpendicular to the page). While these diagrams were devised originally only as simplifying pictorial aids, they do have the capacity to be treated more rigorously when represented as a graph of connected nodes. This approach has been used effectively for structure comparison, by searching for matching sub-graphs [18]; however, this still falls short of a providing a theoretical framework on to which rules could be imposed to specify structural transitions. 

The internal coordinate representation of a contact or adjacency graph is attractive as it eliminates any dependence on a coordinate framework and avoids details of the exact geometry between packed pairs of secondary structure elements. However, as mentioned above, internal representations, being based on adjacent interactions, struggle to capture the long-range chiral features that dominate protein folds. An alternative approach is to retain the essence of a global reference frame in the form of layers of secondary structure. The beta-sheet structure clearly can be treated strictly as a layer, as can a second packed sheet (as in a beta-sandwich) and to a slightly lesser extent, any alpha helices that pack against a sheet will form a reasonable layer. Fortunately, three or four layers comprising 10 to 20 SSEs are sufficient to represent most protein domains. The serious failing of a layer-based scheme is that any structure without a beta sheet cannot be encoded. This omission will be returned to later.

### 2.3. Topology String Representation

Given a layer-based representation, the path of the chain through the layers can be recorded as a string, giving each SSE two coordinates: layer identity and position in the layer. As protein structures do not have an absolute reference frame, some arbitrary conventions must be introduced to assign the coordinate labels. In the system described below, these were that the first point of entry to a layer is assigned position zero with others in the same layer given relative numeric values based on adjacency (which does not imply actual contact in the structure). Layers were given alphabetic labels based on a core of A, B, C, where the middle layer is the beta sheet. Additional alpha and beta layers were designated D and E, respectively. 

Viewing a fold diagram as a coordinate system with the *x*-axis horizontal and the *y*-axis vertical, The *x*-axis (the numeric direction of the layers) was set by taking the first strand in the beta sheet as the origin with strand numbers then increasing left to right. The orientation of the *y*-axis of the diagram was set by assigning the label “A” to the first alpha layer to be occupied and “B” to the first beta layer. The remaining third dimension (z), which runs orthogonal to the “page”’, has only two values depending on whether the SSE runs towards or away from the viewer and are designated “+” and “−”, respectively (resulting in the scheme being referred to occasionally as 2.5 D rather than fully 3D). This scheme thus embodies some of the features of an internal coordinate system but without the loss of chirality. 

Using this system, a sequence of SSEs can be written as a string of coordinates in the form: ZYX.ZYX.ZYX..., such as: +B+0.-A+0.+B-1..., representing a beta-alpha-beta unit. Note however, that the placement of the first beta and alpha on position zero does not necessarily imply that they pack together. More trivially, for every protein chain that begins with a beta strand, this strand will be designated +B+0 and every N-terminal alpha will be +A+0. (The redundant “+0” or “+1” on the “y” coordinate is included only to keep the substrings of equal length for each position which makes reading aligned strings slightly easier). 

**Figure 3:**
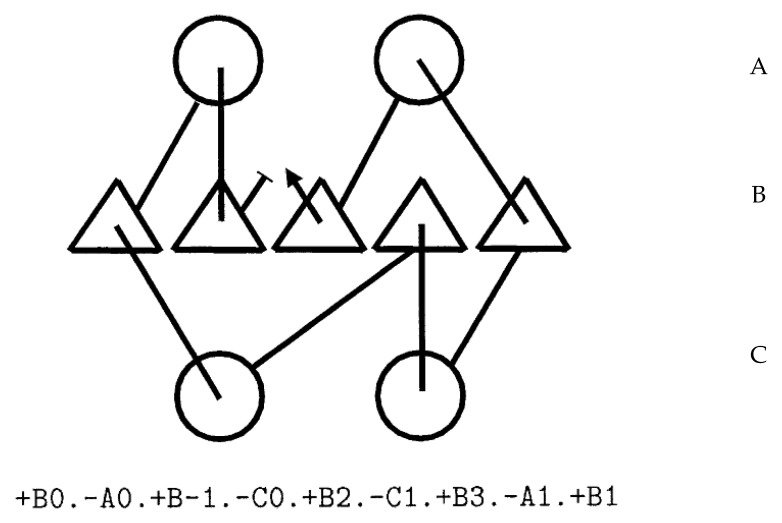
Topology diagram and string. The encoding of a topology diagram into a string is described in the text. In the diagram, helices are circles and strands are triangles. The three secondary structure layers (alpha/beta/alpha) are encoded as A/B/C in the string and secondary structure elements (SSEs) are numbered left to right in each layer.

The beta-alpha-beta unit “+B+0.-A+0.+B-1” specifies a right-handed connection, whereas “+B+0.-A+0.+B+1” is left-handed. Connections ‘below’ the sheet (through layer C) reverse this relationship so “+B+0.-C+0.+B+1” is right-handed, as will a change in “Z” orientation, so “-B-1.+A+0.-B-2” is left-handed. In general: +Bi,-Aj,+Bk is right handed for any k < i. This rule (and its equivalent for other Y-layers and Z-orientation) means that if a process is editing or generating these topology strings, the chirality of the connection can be checked easily and imposed as a constraint. As stated above, this cannot be done in a purely internal coordinate system or quite as easily in a numeric coordinate system of arbitrary orientation. Figure 3 shows an example encoding for a small beta/alpha protein.

### 2.4. Comparing Topology Strings

If the topology strings defined above are to be used for searching or comparison, then because they are not completely free of a reference frame, it is possible for identical substructures to have different strings depending on their position in their structure. However, as the strings depend only on the initial setting of each dimension, there are only four possibilities (±X × ±Y × ±Z)/2 and following the rules outlined above, each string can be written in all frames and any probe sequence compared against them all. (Equivalent to comparing an amino acid sequence against a DNA sequence in all six reading frames). 

Along with a few other constraints, this approach was implemented in a dynamic-programming algorithm to find the largest common substructure (LCS) among topology strings using a metric that minimized the number of edit operations required to transform one string into another [19]. The additional constraints were imposed to maintain the LCS as a compact unit (with no gaps in its beta sheet) and, as such, it can be taken as a competent structure which could represent a common ancestor of both proteins being compared. The resulting LCSs were then further compared to other proteins and to each other and the matrix of relationships represented as a dendrogram that depicts the “evolution” of complex folds from a simple core structure. 

**Figure 4:**
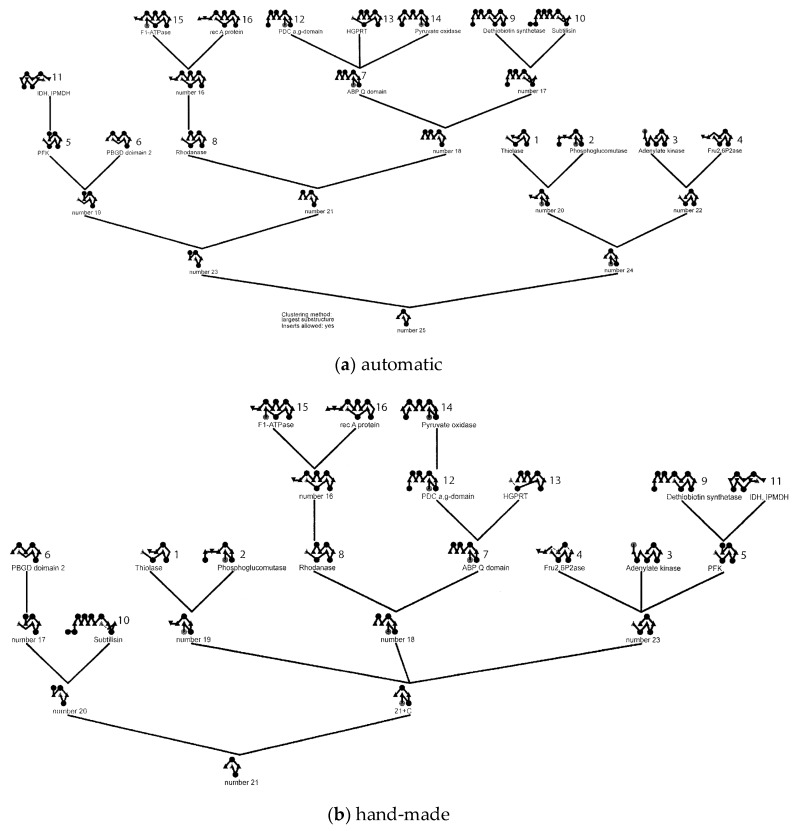
Fold dendrograms for alpha/beta proteins. (**a**) Calculated automatically by finding the largest common substring between topology strings which is compared in part (**b**) with Efimov’s “hand-made’” tree (based on a diagram similar to Figure 1). The 16 known structures used in the calculation are named and numbered while the “ancestral” structure nodes are just labelled as “number X”.

The resulting “tree” is not unlike those developed by Efimov manually and comparing an automatic dendrogram generated for a subset of alpha/beta proteins used in his analysis shows a good correspondence. (Figure 4). A difference between the automatic and manual approaches is that the automatic method generates additional ‘’ancestral” proteins at nodes in the tree which have not been observed (at least at the time Efimov made his trees). In Figure 4, these have simply been assigned arbitrary numbers and have been added to Efimov’s tree to aid comparison. Even with the inclusion of ancestral nodes, it is obvious that some branches could involve multiple steps of SSE additions which are not captured in a single LCS. To investigate the full pathways among protein folds requires a more generative structure-based approach.

## 3. Secondary Structure Lattices

### 3.1. Ideal Forms

Given a lattice of secondary structure layers, it easy to imagine that a constrained random walk along the edges will generate a possible protein structure. The space of folds that are generated will depend on the given layer structure and the connection constraints that are imposed such as chirality and loop cross-overs. These are (so-called) topological constraints, but if the fold is to be realized in 3D, then packing measures are also important, such as compactness (surface/volume ratio or radius of gyration) and SSE packing, especially maintaining a connected beta-sheet or sheets (that is, isolated beta strands are forbidden). 

The space of surface/volume ratios in the context of layer packing was quantified by Finkelstein and Ptitsyn [20,21], and following this, a simpler method can be taken to limit the expected number of secondary structures in each layer. Considering just the number of SSEs in each layer, a small 3 layer alpha/beta/alpha (ABA)-type protein with a single helix packed either side of a 2 strand sheet can be written as: 1-2-1. Since the alpha helix is roughly twice the width of a beta strand, the helices will cover the sheet and similarly for 2-4-2, 3-6-3. Referring to these representations as Forms (as in a pseudo-Platonic sense), then given a number of strands and helices, there will be a limited set of Forms that can allow them to create a compact structure. Given, say, five strands and five helices, then these would be a better (more compact) fit on a 2-5-3 Form rather than a 1-5-4 Form. 

The scope of structures covered by these Forms, besides the three-layer ABA type, also includes the four-layer ABBA type. Then, as an outer layer can be empty (e.g., 0-5-3, 0-5-0), this also includes the 2 layer AB types and a naked sheet. Similarly, from the four-layer ABBA type, we get ABB and BB types. (For clarity, the 4 layer type is written with a “+” between the strand numbers, e.g., 2-4+5-3). All these variations can be arranged systematically with the layers ordered as: ABBA, BBA, BB, B, BA, ABA, so that only a single layer changes with each step. Within each type, the SSEs can also be added so that there is only one change between adjacent Forms; for example, with the ABBA type, starting with the 1-3+3-1 Form we can try and fill the layers starting from the rightmost. However, as mentioned above, adding a helix to give 1-3+3-2, while not impossible, gives an unbalanced packing and it is better firstly to add a strand to the sheet, giving Form 1-3+4-1, before adding a helix to give the 1-3+4-2 Form. 

While this ordering is not based on exact packing energies, the logic behind it is not unlike that observed in the filling of electronic shells by electrons in atoms and if followed systematically, the resulting table of Forms bears a slight resemblance to the periodic table of elements (PToE) [22]. If the ordered layers correspond to Periods and the other dimension corresponds to Groups in the PToE, then the types with fewer layers require gaps while the larger ones fill their additional layers. For example:

1-4+6-1 0-4+6-1 0-4+6-0
1-4+6-2 0-4+6-2 :
1-4+6-3 0-4+6-3 :
2-4+6-3 :    :
2-4+6-3 :    :
2-4+6-3 :    :
1-5+6-1 0-5+6-1 0-5+6-0

Unlike Mendeleev’s wonderful construction, this ordering has no predictive power whatsoever with regards to the properties of proteins but steps between adjacent Forms give some indication of pathways of transitions that might more easily be made through random structural evolution involving destruction or creation of an SSE. Given that there are some large jumps in the current table, it would seem more likely that a more complex multi-dimensional construct would be required to make all single element transitions adjacent.

### 3.2. Generating A Fold-Space

Given a sequence of mixed SSEs that includes at least two beta strands, the set of Forms that can exactly accommodate these SSEs can be selected and starting at every point on each Form (allowing for redundant symmetries), all possible chain paths can be generated in a combinatorial manner (typically, by a depth-first tree transversal). As topology strings can be trivially generated as each path is traced, it is easy to check each move over the Form for violations of connection chirality as well as loop crossings and apply these checks to prevent further progress down the remaining subtree of possible folds. 

**Figure 5:**
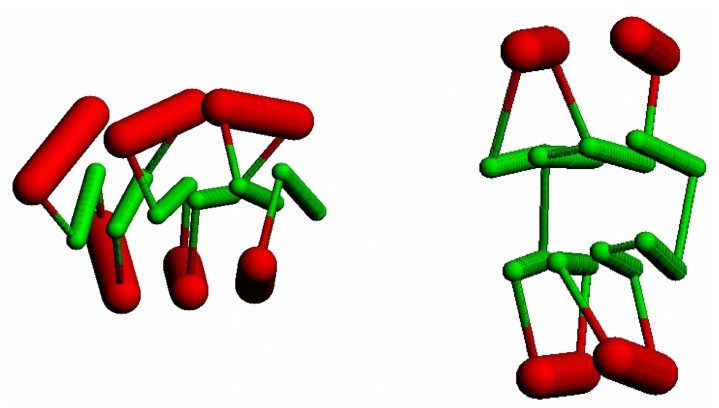
Stick Forms: The “stick” figures derived from a three-layer 3-6-3 (left) and a four-layer 2-4+4-2 (right) Form are shown with strands as green “sticks” and helices as thicker red “sticks”.

The resulting set of protein folds, although only specified in 2.5 D can be trivially mapped onto a “stick” representation of the Form (Figure 5), and it is again a simple matter to then convert the “stick” models to Cα models with loops added as a constrained random walk and the SSEs expanded with ideal geometry. At this point, the nature of the amino acid sequence can be considered with helices orientated about their axis to direct their hydrophobic moment towards the centroid of the model. A similar phasing can be applied to beta-strands but if the sheet is buried, then the phase is relatively arbitrary. Despite these crude approximations, when a sequence is mapped onto the correct Form, the resulting RMSD compared to the native structure is around 5Å over about 100 Cα positions [23]. 

This degree of model accuracy would seem to be sufficient to compare a set of models against a set of known folds (such as a non-redundant collection of domains). However, it was found that at around 5Å RMSD, a transposition of two adjacent beta strands can pass undetected [23], and although the strands may be relatively short, by swapping position, the topology of the structure becomes different. This problem can be avoided by using instead the topology strings directly to identify the fold type as this relies only on string identity and is thus unambiguous. While the model folds are generated from their strings, such a comparison requires that the native structures are also encoded as topology strings. This was done by comparing each “stick” model to every known structure and selecting the best match as a compromise between coverage and fit. Knowing that the topology strings match between a model and native, then the RMSD between them based on this alignment can be accepted as meaningful. 

**Figure 6:**
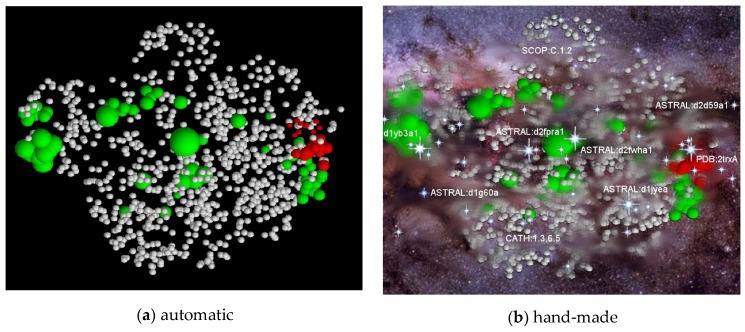
A fragment of fold space: (**a**) The model folds generated from the secondary structure sequence for a small protein is shown with each structure represented as a dot in a projected space (as in Figure 2). The known family from which the SSE sequence was taken is coloured red, and the model structures that correspond to other known folds are coloured green. The size of the coloured spheres is proportional to the number of family members. (**b**) The greater number of novel folds (white) has been likened to the preponderance of dark matter in the Universe.

The comparison of all models to all known domains (or at least a subset with similar structural type) generates a matrix of RMSD values that can be visualised by distance geometry in the same way as described above for a set of all known structures. Interestingly, for a set of known structures with a given Form, the number of model structures that can be generated from this Form (after all topological filters have been applied) greatly outnumbers the known folds by around an order of magnitude (or more for the larger Forms). The visualisation of this fold-space gives the impression that the known folds are embedded in a matrix of unseen folds that has been described, using a cosmological comparison, as the “dark-matter” of protein fold-space [24] (Figure 6).

## 4. Generalised Lattices

### 4.1. Missing Types

Being based on the layer structure imposed by a topologically flat (or open) beta sheet, the Forms described above clearly neglect the large group of all-alpha proteins and other beta-based structures that have closed cylindrical sheets (barrels), circles in 2D, or have helical symmetry, such as beta propellers or beta solenoids or even sheets that do not pack in flat layers, as found in beta prisms. 

**Figure 7:**
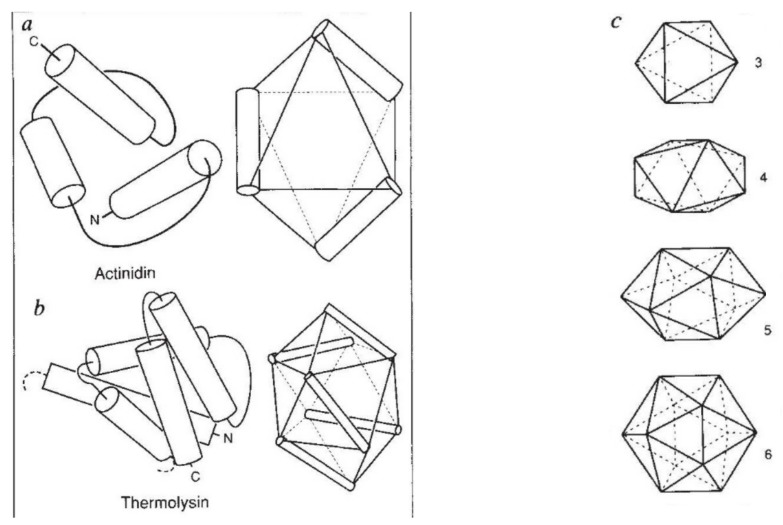
Murzin–Finkelstein polyhedra. (**a**) and (**b**) show how the helices in two all-alpha proteins can be mapped to edges of the solids. (**c**) The set of polyhedra supporting three up to six helices. Including the tetrahedron, which supports two helices (not shown), three of the polyhedra are Platonic solids.

For the all-alpha proteins, the class of convex deltahedra (triangle-faced polyhedra based on packed spheres) devised by Murzin and Finkelstein [25] provides a remarkably accurate way to capture the various helical packing seen in small globular proteins, up to those comprising six helices. (Figure 7) Of their five polyhedra, representing structures from two to six helices, three are Platonic solids. While it is possible to construct customised secondary structure lattices for all the different types of protein, based on an idealisation of their known structures, it would be more satisfying to have a generalised lattice that can capture both the layered types and the globular helical types as well as the beta barrel types and others. 

The layer lattices and the Murzin–Finkelstein (MF) polyhedra share the common building block of an equilateral triangle. If the layer lattice is extended in Z (into the “page”) and twisted, then the Z-extension end-points can be sufficiently displaced to form equilateral triangles as they do in the MF solids. This geometry can be attained for the helical layers adjacent to the sheet with the sheet having a simple twist (with strands at half-helical spacing). This provides a good lattice for the beta/alpha class (Figure 5) and allows additional helical domains, represented by MF polyhedra to be added to the surface. While providing useful extensions, the result is far from a general solution.

The twisted layer lattice for the 2-5-2 Form consists of two hexagons with a 30° relative rotation and as such is a fragment of two layers of a close-packed hexagonal (CPH) lattice and also the face-centred cubic (FCC) lattice. (This can be seen embedded in the 3-6-3 form shown in Figure 5). As MF polyhedra also include two fragments of this lattice in the tetrahedron and octahedron, it is interesting to consider whether a sufficient approximation of both MF solids and layers could both be found in the hexagonal based or other lattices. While this route offers some progress for smaller proteins, for larger structures, extending the beta sheet leads to increasing distortions. For all helical proteins, a wide variety of interactions can be supported but care needs to be taken when the helices are extended to their native lengths as clashes between the ends can easily be created—which is a problem neatly avoided in the convex MF polyhedra. This problem does not arise with the more co-linear helices found in transmembrane proteins and a hexagonal-based lattice provides a good framework for this class [26]. 

An alternative regular lattice, not unrelated to those based on hexagonal symmetry, can be found in the rhombic dodecahedron, which is a convex polyhedron with 12 congruent rhombic faces. Allowing a connection across the shorter diagonal of each rhombus allows it to be treated as an additional MF polyhedron with 24 half-faces and the capacity to support 7 helices. The rhombic dodecahedron is also able to pack in a lattice and fill-space, which opens the potential for further expansion. However, this lattice can be viewed as layers of slightly distorted hexagons (with diameters in the ratios 2:√3:√3) and as such offers little over the FCC or CPH lattices discussed above.

### 4.2. Deltahedra and Tetrahedron “Packing”

The accretion of spheres around a tetrahedral core, with each new addition making three contacts, generates a series of polyhedra referred to as Deltahedra (or more generally as close-packed clusters) which have been well studied in the context of metal clusters. These shapes, which are often fragments of regular hexagonal lattices, provide good approximations to most of the MF polyhedra but without including the square packings found in the octahedron, snub-disphenoid, and gyroelongated square bipyramid (supporting 3, 4, and 5 helices). 

**Figure 8:**
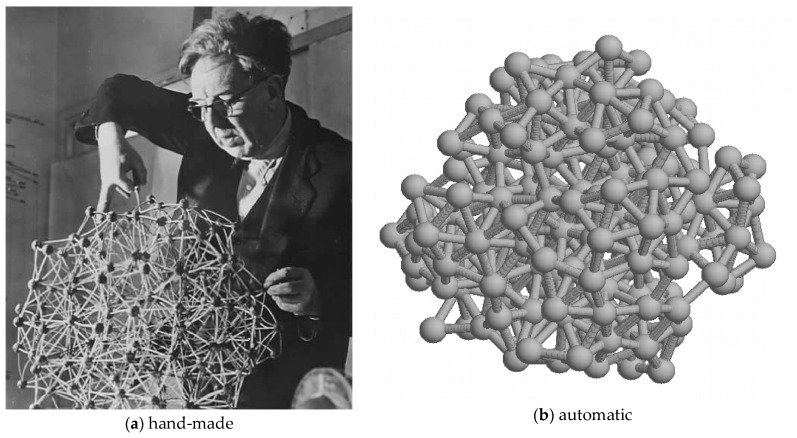
A Bernal lattice: (**a**) J. D. Bernal in the 1960s working on his model for liquid water based on tetrahedral “packing”. Since tetrahedra cannot tile space, this is a pseudo-lattice incorporating many discontinuities (hence the liquid nature of water). (**b**) A more recent computer-generated version (made with a program written by the author).

Extending this process leads to an irregular lattice that has been taken as a model for amorphous solids and liquids, particularly water [27]. Since tetrahedra cannot pack to fill space, discontinuities arise between sub-domains, however, these are often small and if the spheres are considered to be slightly soft (or the bonds between them flexible) then quite extensive pseudo-lattices can be created. Originally developed by J. D. Bernal, this pseudo-lattice will be referred to as a Bernal lattice (Figure 8).

### 4.3. Penrose Tiling and Quasi-Lattices

An elegant way to avoid the distortions of the tetrahedral “lattice” and the over-regularity of the hexagonal lattices is to consider the quasi-lattices found in crystals with (forbidden) five-fold symmetry. This paradox of a crystal with 5 fold symmetry was resolved by Alan MacKay (a student of Bernal’s) and others with the construction of quasi-lattices based on a 3D Penrose tiling. In the canonical form, two regular solids (an oblate and prolate golden rhombohedron) combine to fill space in such a way that local 5 fold symmetry can be found but without regular repetition. 

The core element of this is the rhombic triacontahedron which has 30 (triaconta in Greek) rhombic faces with 60 edges and 32 vertices or 44 vertices if the internal points of the rhombi are included. As the core still remains slightly hollow, the centroid can be included giving 45 vertices. This is more than enough to support most moderate protein domains and the added complexity of extending the lattice with additional rhombohedra is not needed.

### 4.4. Chain Tracing over A Lattice

The layer Forms and MF solids considered above covered a range of sizes such that a Form can be selected to exactly fit a given chain of secondary structures. This provided a powerful constraint on the number of possible chain paths compared to those that could be generated on an open (or unbounded) lattice. In addition, for the alpha/beta class, each layer of the selected Form was designated as a particular secondary structure type, again greatly reducing the possible paths.

With a general lattice these constraints do not exist which, besides opening the solutions to structures that are not compact, also means that the secondary structure type of an edge must be defined “on-the-fly” in a consistent way. As previously suggested by Murzin, any edge on a lattice can be designated as “beta” by allowing the double passage of the chain (like a double bond in chemistry) and it can be left to later post-processing to find if these “bonds” can be regularised into a connected sheet. Although never tested, resolving the geometry of strands only after fold generation would delay the specification of sheet topology (depending on the direction the strands are parted) which would, in turn, delay checks on their connection chirality and without chiral constraints, the possible chain paths would increase greatly.

**Figure 9:**
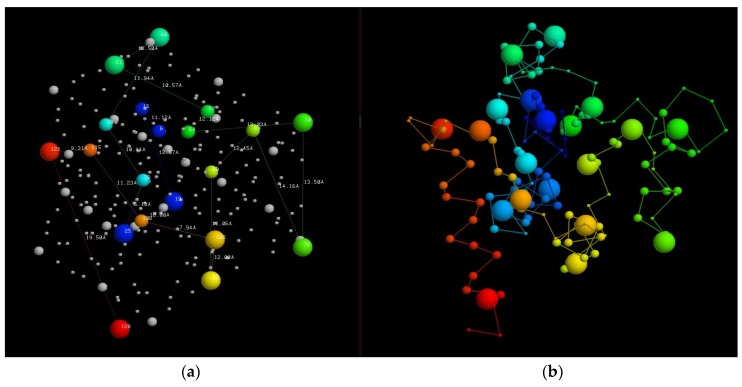
An augmented Bernal lattice: (**a**) a fragment of a Bernal lattice (large dots) is augmented with an additional pair of points along each edge (small dots). Vertices are selected (coloured) to represent the path of a protein chain (**b**) with helices on lattice edges and strands along lines connecting satellite points. Cα atom positions are marked by small spheres which are linked by a fine line. Points are coloured blue to red following the chain from its amino to carboxy terminus.

Alternatively, the geometry of adjacent strands can be assessed during the enumeration process. This was done by expanding each vertex into a set of vertices with points being added along each edge one-third of the way towards all connected vertices. To pass along an edge, a beta strand must select a pair of end-points that has compatible geometry with any strand already on the same edge or on an adjacent connected edge. A rough score was devised combining the left-hand twist expected between adjacent strands while still keeping the strands reasonably aligned (Figure 9).

### 4.5. Testing Different Lattices

A computer program was written to take any lattice and identify edges that can be traversed and on each of these, the satellite points to support beta strands were added. Models were roughly scored and ranked based on the packing interactions made by their edges and the better packed models were then expanded to an alpha carbon model with secondary structures comprising the number of residues specified in the initial sequence and loops added as a pseudo-random walk between vertices, again incorporating the number of residues initially specified in the sequence.

**Figure 10:**
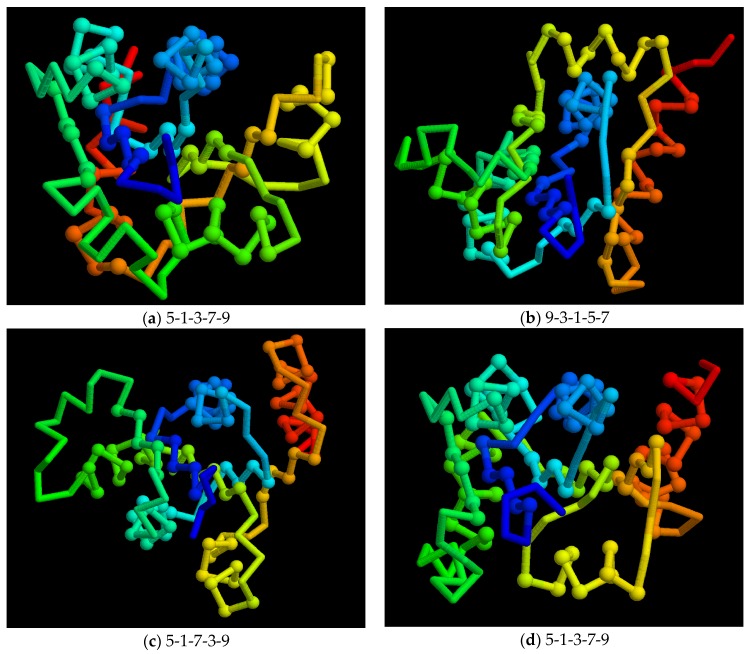
Model folds: A small sample of model folds is shown as a Cα backbone trace (as in Figure 9b) with the chain coloured blue to red corresponding to amino to carboxy terminus. Residues in secondary structures are marked as a small sphere. The order of the strands in the sheet is shown below each fold. Note that even though folds (**a**) and (**d**) have the same strand order, the strand direction is not recorded and these models have different folds.

Initial tests were performed on both the tetrahedral-based (Bernal) “lattice” and the rhomboid-based (Penrose) quasi-lattice. The former was limited to approximately convex (deltahedral) clusters up to 25 points and for the latter, a single triacontahedron was used. Both lattices contained sufficient geometric variation to capture twisted beta sheets of several strands and a selection of beta/alpha models are shown in Figure 10. 

While it is encouraging that open beta sheets can be captured, the motivation to move to a more general lattice was to combine the open and barrel sheets in a single framework. For the Bernal lattice, twisted 6 strand barrels can easily be found based on the ubiquitous 3 fold faces of the tetrahedra but although the lattice has many 5 fold centres, the spacing around these is too wide for strands in a sheet. However, omitting a strand from the 6 strand barrel and connecting the neighbouring strands at a greater angle closes the gap enough to produce a good 5 strand barrel. 

With the lack of any 4 fold centres, locating the common 8 strand barrels might seem more problematic, but a saddle-shaped rhombus formed from the faces of two adjacent tetrahedra can be matched in pairs to generate a twisted 8 strand barrel. As with five, 7 strand barrels can be formed by omitting a strand. The 5 fold axis (across a slightly compressed icosahedron) allows a completely symmetric 10 strand barrel to be constructed and again by omitting a strand, also a 9 strand barrel. With the exception of the transmembrane porins, such large barrels are rare. 

**Figure 11:**
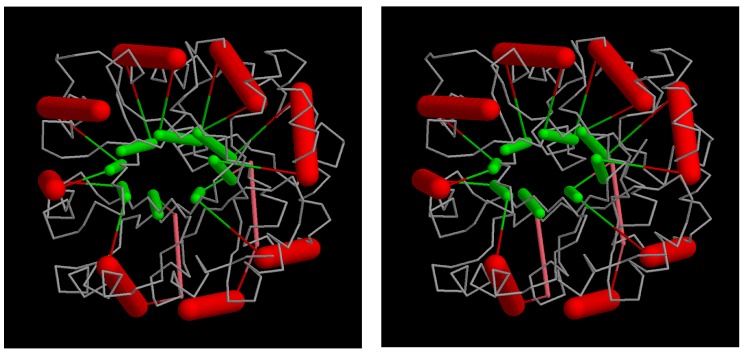
TIM in a triacontahedron: the beta/alpha barrel structure of triosephosphate isomerase (PDB:1timA) is mapped into the core of a triacontahedron. Although the SSEs are in roughly the right positions, their relative angles are not ideal. The parts comprise a “cross-eyed” stereo pair.

Similar relationships can be found in the triacontahedron as it has many triangular and rhombic faces as well as 5 fold centres of symmetry. However, the analogous frameworks for beta barrels in this lattice results in parallel strands that do not capture the twist between strands in a barrel. A pair of rhombi with a skewed juxtaposition can, however, be found in the centre of the triacontahedron and based on this, a plausible 8 strand beta/alpha barrel can be traced that has a reasonable fit to the canonical barrel in triosephosphate isomerase, although the angles between the SSEs are not ideal. (Figure 11). Extending the quasi-lattice has the potential to generate additional relationships between edges but these were not investigated.

## 5. Conclusions

An extended pseudo-lattice generated through the accretion of tetrahedra can be used to capture both open-twisted beta sheets and beta barrels, and the dislocations implicit in trying to pack tetrahedra do not become a serious problem over the range of sizes encountered in typical protein domains up to around 300 residues. Some gentle regularisation can also be applied to anneal interfaces where the vertices of adjacent tetrahedra come sufficiently close to be fused. Beyond this domain size, larger dislocations may indeed be an advantage as they break the lattice into domain-sized connected regions. 

The adoption of a more extended lattice raises difficulties when it comes to the enumeration of possible chain paths as these are much more numerous than those allowed over a small polyhedron or Form that exactly fits the chain. Indeed, the numbers grow to the extent that complete enumeration is impossible. This places increased onus on the application of folding and packing constraints that can terminate chain extensions that lead to chiral violations or are not sufficiently compact. However, if the edges are traversed in the order that their tetrahedra were added to the core, then the choice of direction for each next step will initially explore edges that lie towards the centre. 

Although computational demand remains heavy even for small proteins, a fast string-based method to quickly evaluate the packing of each SSE as it is added has been developed that can allow almost a billion chain paths to be evaluated on a moderate laptop in less than an hour (without any parallel calculations). Since the resulting models have come from an irregular lattice, they cannot be filtered for symmetrically equivalent structures in the same way as those generated symbolically on the ideal Forms but the extraction of the sheet topology provides a step towards this. If the models are being used for decoy generation, such variations may be acceptable but to extract unique folds requires a more detailed superposition-based comparison.

This review has focused only on small fragments of fold space with examples given for folds generated from a single secondary structure string consisting of around ten SSEs. Even in this small corner, the number of possible folds, under the current constraints, is of the order of 1000—as can be estimated from the number of points in Figure 6a. This exercise can be repeated for every permutation of the alpha and beta elements in that string and again repeated for every possible mixture of SSEs over any length up to, say, roughly 20 elements in a reasonably large domain. In theory, the current Bernal lattice and current filters can be used to do this but the number of models thus generated would be vast and take some considerable time to analyse and classify. 

If the current approach were to have any particular use, it would probably be in applications that involve a more restricted exploration, such as limiting the range of variation to a particular sub-region, maybe on the edge of a domain, or to permute edges over adjacent free vertices in the lattice to explore pathways of divergence from a starting structure. Combining this with the Efimov-like “trees” described above could provide a mechanism for exploring the routes of “evolutionary” transitions from one fold to another. This approach could also be used to explore how a simple core could expand into a wider family but following this route, it would also be good to include the process of gene duplication. 

Given that the space of possible folds seems to be so vast, a major puzzle is why the number of observed folds is so small in comparison (maybe around a few 1000). The most likely explanation is that the simple filters applied in the generation of the model folds are incomplete and may also interact in complex ways. Despite an attempt to quantify these factors [28], it may not be possible to fully enumerate them, especially as there are thermodynamic and kinetic based factors that can impose a strong selection on the types of fold that are favoured and accessible [29,30]. Recent developments in deep learning may provide a more flexible mechanism to better capture all such contributions, but on the other hand, this approach may simply constrain solutions to recapitulate the known folds used in the training set. 

An alternative explanation is that Evolution has simply “made do” with elaborations based on a few primitive folds that arose by chance in the earliest prebiotic times. This frozen accident view might be explored by the methods described above but only in outline terms, as it would seem to be rather unlikely that the set of known folds could be predicted, literally, ab initio from conditions that prevailed over three and a half billion years ago.
